# Fusing Infrared and Visible Images of Different Resolutions via Total Variation Model

**DOI:** 10.3390/s18113827

**Published:** 2018-11-08

**Authors:** Qinglei Du, Han Xu, Yong Ma, Jun Huang, Fan Fan

**Affiliations:** 1 Electronic Information School, Wuhan University, Wuhan 430072, China; dql822@163.com (Q.D.); xu_han@whu.edu.cn (H.X.); mayong@whu.edu.cn (Y.M.); junhwong@whu.edu.cn (J.H.); 2 Air Force Early Warning Academy, Wuhan 430019, China

**Keywords:** image fusion, different resolutions, total variation, infrared

## Abstract

In infrared and visible image fusion, existing methods typically have a prerequisite that the source images share the same resolution. However, due to limitations of hardware devices and application environments, infrared images constantly suffer from markedly lower resolution compared with the corresponding visible images. In this case, current fusion methods inevitably cause texture information loss in visible images or blur thermal radiation information in infrared images. Moreover, the principle of existing fusion rules typically focuses on preserving texture details in source images, which may be inappropriate for fusing infrared thermal radiation information because it is characterized by pixel intensities, possibly neglecting the prominence of targets in fused images. Faced with such difficulties and challenges, we propose a novel method to fuse infrared and visible images of different resolutions and generate high-resolution resulting images to obtain clear and accurate fused images. Specifically, the fusion problem is formulated as a total variation (TV) minimization problem. The data fidelity term constrains the pixel intensity similarity of the downsampled fused image with respect to the infrared image, and the regularization term compels the gradient similarity of the fused image with respect to the visible image. The fast iterative shrinkage-thresholding algorithm (FISTA) framework is applied to improve the convergence rate. Our resulting fused images are similar to super-resolved infrared images, which are sharpened by the texture information from visible images. Advantages and innovations of our method are demonstrated by the qualitative and quantitative comparisons with six state-of-the-art methods on publicly available datasets.

## 1. Introduction

Compared with information provided by a single sensor image, multi-sensor images can supply additional information with feature complementarity for complex and complete scene representations and improved visual understanding. By integrating useful information from different sensor images, we can generate a single composite image, which is informative and suitable for follow-up processing and decision-making. In particular, infrared and visible image fusion is an indispensable branch that plays an important role in military and civilian applications [1] and has been used to enhance the performance in terms of human visual perception, object detection, and target recognition [2,3,4,5,6,7,8].

Visible sensors can capture reflected light to provide background details, such as vegetation, texture, area, and soil with high spatial resolution because they operate in the same waveband that can be recognized by human vision [9]. However, under unsatisfactory light conditions, scene information is lost. By contrast, infrared sensors map thermal radiation emitted from objects to gray images; therefore, these sensors exhibit unique advantages in overcoming adverse light conditions and highlighting thermal targets, such as pedestrians and vehicles. Nevertheless, due to limitations of hardware and environments, infrared images are often accompanied by blurred details, serious noise, and considerably low resolution. These limitations encourage the fusion of infrared and visible images to produce a single image, which can simultaneously highlight thermal radiation for target detection and reserve texture information for characterization of appearance.

According to different representations and applications, the fusion process can be conducted at the following three levels [10]: pixel, feature, and symbol levels. Pixel-level fusion is on the lowest level and fuses different physical parameters. Feature-level fusion involves feature descriptor, probability variables, and object labels. Symbol-level fusion uses high abstraction of information, such as representative symbols or decisions, for fusion. The focus of this paper is on pixel level. From another perspective, existing infrared and visible image fusion methods are divided into eight categories according to their corresponding theories [1], including multi-scale transform-based methods [11,12], sparse representation-based methods [13,14], neural network-based methods [15,16], subspace-based methods [17,18], saliency-based methods [19,20], hybrid methods [21,22], deep learning-based methods [23,24], and other fusion methods [10,25].

**Figure 1:**
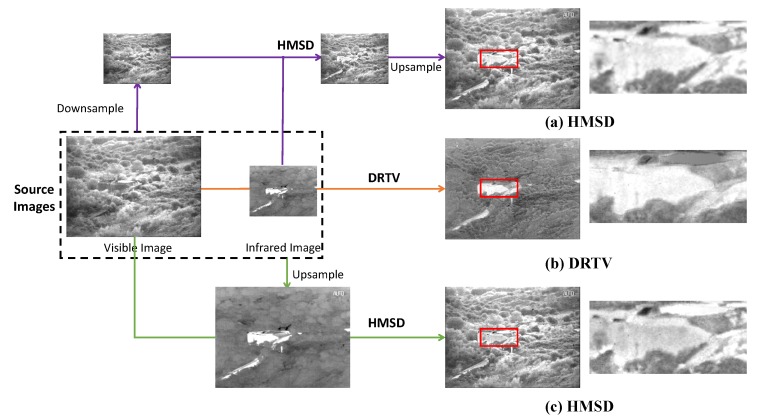
A typical example for fusing infrared and visible images of different resolutions through (**a**) downsampling the visible image; (**b**) our method; and (**c**) upsampling the infrared image.

Despite the notable progress of image fusion, fusing infrared and visible images of different resolutions remains a challenging task. Infrared images constantly suffer from considerably lower resolution compared with visible images. Under this condition, if existing methods are applied to fusing source images, then eliminating resolution differences, through downsampling visible images or upsampling infrared images as an example, is the first problem to solve. As shown in Figure 1, to intuitively illustrate the fusion process, we take a typical image pair and a typical fusion method, such as hybrid multi-scale decomposition (HMSD) [26], as an example. In procedure (a), the visible image is downsampled before using as a source image for fusion, and the fused image is upsampled considering consistency and comparability. Although the fused image can be forcibly converted into high resolution, the discarded high-quality texture information in the visible image caused by downsampling operation by image processing cannot be retrieved from the viewpoint of information theory; hence, the clarity of the fused image is not improved. In procedure (c), the original infrared image is upsampled to high resolution before fusion. As shown in the figure, upsampling results in blur and inaccuracy in the infrared image, which is also fused into the resulting image; hence, the fused image in procedure (c) is accompanied by serious noise. In addition, the principle of existing fusion rules typically focuses on preserving texture details in source images. This condition may be problematic in the fusion of infrared and visible images because infrared thermal radiation information is characterized by pixel intensities and usually contains few texture details that may fail to be fused in the resulting image. However, such information is often crucial to highlight the targets. This can be shown in procedures (a) and (c), in which the fused image loses the property of high contrast similar to that in the infrared image, submerging the target in the clutter background.

To address the above-mentioned challenges, in this paper, we propose a new algorithm termed as Different Resolution Total Variation (DRTV) for infrared and visible image fusion. In particular, we formulate the fusion problem as a convex optimization problem characterized by total variation model and seek the optimal fused image that minimizes an energy function. This formulation accepts different resolutions of infrared and visible images. The energy consists of a data fidelity term and a regularization term. The data fidelity term constrains that the downsampled fused image should present similar pixel intensities with the original infrared image. The regularization term ensures that the gradient distribution in the visible image can be transferred into the fused image. The two terms can guarantee that our fusion result contains the thermal radiation information from the infrared image and the texture details from the visible image. To optimize the solution procedure, we apply the fast iterative shrinkage-thresholding algorithm (FISTA) [27] framework to accelerate convergence, e.g., the convergence speed is increased to $O\left(1/k^{2}\right)$ with *k* being the iteration number. Owing to the preceding causes, the cost for acquiring high-quality infrared and visible fused images can be considerably reduced, thereby providing substantial benefit to military equipment and civilian industries relying on infrared and visible image fusion. For intuitive comparison, Figure 1b shows the fused result of DRTV on the same source image pair, where we zoom in a small region in the red box for clear comparison. Figure 1 shows that the fused image generated by our DRTV, i.e., Figure 1b, is clearer with less noise compared with Figure 1a,c due to the forced resolution conversion. In addition, our fusion result looks like a super-resolved infrared image, which preserves the property of high contrast in the infrared image, clearly highlighting the target.

The contributions of this paper include the following two aspects. On the one hand, the proposed DRTV makes a breakthrough in fusing visible and infrared images of different resolutions, which can considerably prevent loss of texture information caused by compression or blur and inaccuracy due to forced upsampling, without expenses on upgrading hardware devices. On the other hand, the fused images obtained by DRTV visually benefit from rich texture information; simultaneously, the thermal targets are easily detected. By changing the regularization parameter in our model, we can subjectively adjust the visual similarity between the fused image and visible/infrared image.

The remainder of this paper is organized as follows. Section 2 describes the problem statement and formulation for infrared and visible image fusion. Section 3 provides the optimization strategy of our algorithm. In Section 4, we compare DRTV with several state-of-the-art methods on publicly available datasets and analyze the results. Conclusions and future works are presented in Section 5.

## 2. Problem Statement and Formulation

We denote $\mathrm{Vis}\in R^{m\times n}$ the visible image of size m×n and $\mathrm{IR}\in R^{\frac{m}{c}\times \frac{n}{c}}$ the infrared image of size $\frac{m}{c}\times \frac{n}{c}$, where *c* is a constant denoting the ratio between the infrared image resolution and the resolution of the visible image. $X\in R^{m\times n}$ denotes the fused image. Note that the size of X is m×n rather than $\frac{m}{c}\times \frac{n}{c}$.

Our goal for the given Vis and IR is to fuse them to generate a high-resolution fused image, which should virtually retain important information in source images, e.g., thermal radiation information in the infrared image, and texture features in the visible image.

### 2.1. Data Fidelity Term

Previous methods generally apply a restriction on source images, in which visible and infrared images should share the same resolution. When this restriction is not satisfied, downsampling the visible image is one solution, but the information translated into the fused image also is similarly limited. Employing the upsampled infrared image as the prior knowledge to maintain thermal radiation information is another solution. However, thermal information after upsampling is constantly blurred and inaccurate. If we constrain fused information to follow the blurred source, then the fused result is even less accurate. Therefore, we assume in this study that the fused image after downsampling possesses similar pixel intensities to the original infrared image. Least squares fitting is used to model this relationship:

(1) $$\varepsilon_{1}\left(X\right)=\frac{1}{2}{∥\psi X-\mathrm{IR}∥}_{F}^{2},$$

where ${∥\cdot ∥}_{F}$ denotes the Frobenius norm of matrix, which is defined as ${∥X∥}_{F}=\sqrt{\sum_{i,j}X_{i,j}^{2}}$. ψ denotes the downsampling operator of the nearest interpolation. If the high-resolution image is defined as $S_{M\times N}$ and the downsampled image is $O_{m\times n}=\psi S_{M\times N}$, then the operator ψ can be denoted as $O_{i,j}=S_{\left[i\times \frac{M}{m}\right],\left[j\times \frac{N}{n}\right]}$, where $\left[\cdot \right]$ represents rounding the data to the nearest integer. $\varepsilon_{1}\left(X\right)$ is a data fidelity term. According to the preceding constraint, the thermal target remains prominent in the fused image.

### 2.2. Regularization Term

The regularization term is essentially the prior knowledge used to avoid overfitting of least squares. One requirement is to allow thermal targets in the fused image to remain noticeable. Additional detailed appearance information in the visible image is expected to be preserved in the fused result. The direct approach is to constrain similar pixel intensity distribution in the fused and visible image. However, the same constraint is applied in $\varepsilon_{1}\left(X\right)$, and pixel intensity distributions of infrared and visible images considerably vary due to their different manifestations. Therefore, this approach is contradictory, and we attempt to search another feature to characterize appearance information in the visible image.

According to the human visual perception system, image edges and textures are abundant when the spatial frequency is large. Moreover, the spatial frequency is a metric based on gradient distribution. Understanding that the detailed appearance information is basically represented by gradient distribution of the image is easy. On the basis of this observation, we require that the fused image should present similar gradients with the visible image, which is represented by the $\ell_{p}$ norm (p≥0):

(2) $$\varepsilon_{2}\left(X\right)={∥\nabla X-\nabla \mathrm{Vis}∥}_{p},$$

where ∇ is the gradient operator and $\varepsilon_{2}\left(X\right)$ should be as small as possible.

By using $\nabla_{i,j}=\left(\nabla_{i,j}^{h},\nabla_{i,j}^{v}\right)$ to represent the image gradient at pixel (i,j) and $x_{i,j}$ to denote the pixel of the *i*-th row and *j*-th column in the image, we obtain the following:

(3) $$\begin{array}{c} \nabla_{i,j}^{h}x=x_{i,j}-x_{i,j+1},\nabla_{i,j}^{v}x=x_{i,j}-x_{i+1,j}, \end{array}$$

(4) $$\begin{array}{c} \left|\nabla_{i,j}x\right|=\sqrt{{\left(\nabla_{i,j}^{h}x\right)}^{2}+{\left(\nabla_{i,j}^{v}x\right)}^{2}}. \end{array}$$

If *i* is the last row of the image, then we set $\nabla_{i,:}^{v}x=0$. Similarly, if *j* is the last column, then we set $\nabla_{:,j}^{h}x=0$.

We now consider the $\ell_{p}$ norm in $\varepsilon_{2}\left(X\right)$. Given that the image intensities are often piece-wise smooth across the image domain, their gradients tend to be sparse, and the non-zero elements usually correspond to the boundaries [28,29]. For constraining the fused image to exhibit similar pixel gradient distribution with the visible image, the differences of gradients between the images should be as sparse as possible; that is, the number of non-zero entries of gradient difference should be as small as possible. This condition is theoretically equivalent to minimizing the $\ell_{0}$ norm of gradient difference, i.e., p=0. However, we must traverse all cases to obtain the $\ell_{0}$ norm of a matrix; hence, obtaining the $\ell_{0}$ norm is non-deterministic polynomial-time hard (NP-hard). Fortunately, for vectors, the $\ell_{1}$ norm is the optimal convex approximation of the $\ell_{0}$ norm and can be easily solved. The restricted isometry condition [30] also theoretically guarantees the exact recovery of sparse solution by $\ell_{1}$ norm. For a vector $v\in R^{mn\times 1}$, the $\ell_{1}$ norm is defined as ${∥v∥}_{1}=\sum_{i=1}^{mn}\left|v_{i}\right|$. Specifically, if the energy function is the $\ell_{1}$ norm of the gradient, it is a total variation model [10,31]. If $\varepsilon_{2}\left(X\right)$ is calculated by a norm of vectors, we can directly set the $\ell_{p}$ norm as $\ell_{1}$ norm. However, in Equation (2), $\varepsilon_{2}\left(X\right)$ is the norm of the matrix. For the matrix ∇X−∇Vis, if the optimal convex approximation of vectors above is to be applied, the norm of the matrix should be equal to the sum of absolute values of all elements. Note that ${∥\nabla X-\nabla \mathrm{Vis}∥}_{p}$ is a norm on the gradient and the discrete isotropic TV-norm [32], i.e., ${∥\cdot ∥}_{TV_{I}}$ is defined by the sum of gradients of all elements, which is defined as:

(5) $${∥x∥}_{TV_{I}}=\sum_{i=1}^{m-1}\sum_{j=1}^{n-1}\sqrt{{\left(x_{i,j}-x_{i+1,j}\right)}^{2}+{\left(x_{i,j}-x_{i,j+1}\right)}^{2}}+\sum_{i=1}^{m-1}\left|x_{i,n}-x_{i+1,n}\right|+\sum_{j=1}^{n-1}\left|x_{m,j}-x_{m,j+1}\right|,$$

where x is a matrix of size m×n. It motivates us to convert the minimization of the $\ell_{1}$ norm to that of the TV-norm.

In addition, another important advantage of the regularization based on ${∥\cdot ∥}_{TV_{I}}$ is its insensitivity to outliers, which always correspond to sharp edges in image processing. Therefore, we use ${∥\cdot ∥}_{TV_{I}}$ to define $\varepsilon_{2}\left(X\right)$ as follows:

(6) $$\varepsilon_{2}\left(X\right)={∥X-\mathrm{Vis}∥}_{TV_{I}}.$$

### 2.3. Formulation of the Fusion Problem

Taking comprehensive consideration of $\varepsilon_{1}\left(X\right)$ and $\varepsilon_{2}\left(X\right)$, positive regularization parameter λ is introduced herein to control the trade-off between the data fidelity term and the regularization term. Then, the fusion problem can be expressed as follows:

(7) $$X=\arg\min_{X}\;\left\{\varepsilon \left(X\right)=\varepsilon_{1}\left(X\right)+\lambda \varepsilon_{2}\left(X\right)\right\},$$

which is equivalent to:

(8) $$X=\arg\min_{X}\;\left\{\varepsilon \left(X\right)=\frac{1}{2}{∥\psi X-\mathrm{IR}∥}_{F}^{2}+\lambda {∥X-\mathrm{Vis}∥}_{TV_{I}}\right\}.$$

Thus far, the fusion problem is converted to solve the fused image X in Equation (8), which minimizes the energy function $\varepsilon \left(X\right)$.

## 3. Optimization

The optimization problem (8) is clearly a convex optimization problem; therefore, a global optimal solution that minimizes $\varepsilon \left(X\right)$ exists. In our model, the data fidelity term is smooth, while the regularization term is non-smooth; thus, we can conclude that problem (8) is in the form of:

(9) $$X=\arg\min_{X}\;\left\{F\left(X\right)=f\left(X\right)+g\left(X\right)\right\},$$

where *f* is a smooth convex function, of which the gradient is Lipschitz continuous, and *g* is a continuous, but possibly non-smooth, convex function. Specifically,

(10) $$\begin{array}{c} f\left(X\right)=\frac{1}{2}{∥\psi X-\mathrm{IR}∥}_{F}^{2},\;g\left(X\right)=\lambda {∥X-\mathrm{Vis}∥}_{TV_{I}}. \end{array}$$

The iterative shrinkage-thresholding algorithm (ISTA) [33] can be used to resolve the preceding problem. Although ISTA can solve general convex optimization problems, its convergence rate is proven to be only $O\left(1/k\right)$ with *k* being the iteration number. For optimizing the slow convergence of ISTA, *Beck* and *Teboulle* proposed FISTA [27]. Its convergence rate can be improved to $O\left(1/k^{2}\right)$. The iterative step in FISTA can be rewritten as follows:

(11) $$X^{k}=\arg\min_{X}\;\left\{\frac{L}{2}{∥X-\left(Y^{k}-\frac{1}{L}\nabla f\left(Y^{k}\right)\right)∥}_{F}^{2}+\lambda {∥X-\mathrm{Vis}∥}_{TV_{I}}\right\}.$$

We denote $\psi^{T}$ as the inverse operation of ψ and set $Y=Y^{k}-\psi^{T}\left(\psi X^{k-1}-\mathrm{IR}\right)/L$, where *L* is a Lipschitz constant of $\psi^{T}\left(\psi X^{k-1}-\mathrm{IR}\right)$, which can be set as 1 herein. By approximating $\nabla f\left(Y^{k}\right)$ with $\nabla f\left(X^{k-1}\right)$ in the FISTA framework, the formula (11) then can be rewritten as:

(12) $$X^{k}=\arg\min_{X}\;\left\{\frac{L}{2}{∥X-Y∥}_{F}^{2}+\lambda {∥X-\mathrm{Vis}∥}_{TV_{I}}\right\}.$$

According to the standard FISTA framework, we can optimize Equation (8) by using Algorithm 1. From the iteration process, each iterate of FISTA depends on the previous two iterates and not only on the last iterate as in ISTA. Therefore, FISTA can achieve a fast convergence rate.
**Algorithm 1:** FISTA framework for DRTV.**Input:** L=1, λ, $t_{1}=1$, $X^{0}=\psi^{T}\mathrm{IR}$, $Y^{1}=\psi^{T}\mathrm{IR}$**Output:** X1:**for**k=1**to***Maxiteration***do**;2:   $Y=Y^{k}-\psi^{T}\left(\psi X^{k-1}-\mathrm{IR}\right)/L$;3:   $X^{k}=\arg\min_{X}\;\left\{\frac{L}{2}{∥X-Y∥}_{F}^{2}+\lambda {∥X-\mathrm{Vis}∥}_{TV_{I}}\right\}$;   \\ solved by Algorithm 2;4:   $t_{k+1}=\left[1+\sqrt{1+4{\left(t_{k}\right)}^{2}}\right]/2$;5:   $Y^{k+1}=X^{k}+\frac{t_{k}-1}{t_{k+1}}\left(X^{k}-X^{k-1}\right)$;6:**end for**;7:X is obtained as $X^{k}$ in the last iteration.

To solve the optimization problem in Line 3 in Algorithm 1, we set Z=X−Vis. Then, we obtain the following minimization problem:

(13) $$Z^{k}=\arg\min_{Z}\;\left\{\frac{L}{2}{∥Z-\left(Y-\mathrm{Vis}\right)∥}_{F}^{2}+\lambda {∥Z∥}_{TV_{I}}\right\}.$$

The minimization problem in Equation (13) is the problem of total variation (TV) minimization [31], and $X^{k}$ can be updated by $Z^{k}+\mathrm{Vis}$. The first term reflects that Z should be similar to Y−Vis in terms of content. The second term, i.e., the TV term, is a constraint on the piece-wise smoothness of Z. The smooth constraint has been proven to allow the existence of step edges; hence, the boundary is not blurred when minimizing the energy function [34].

We follow the standard procedure [32,35] for solving the TV minimization problem in Equation (13) and summarize the entire procedure in Algorithm 2. The convergence rate is also considerably superior to other methods based on gradient projection. Refer to [32] for the detailed derivation. For ease of understanding, we use the same symbol notations as those in [36]. The linear operator is defined as: $L{\left(R,S\right)}_{i,j}=R_{i,j}-R_{i-1,j}+S_{i,j}-S_{i,j-1}$. The corresponding inverse operator is presented as follows: $L^{T}\left(X\right)=\left(R,S\right)$, where $R_{i,j}=X_{i,j}-X_{i+1,j}$, $S_{i,j}=X_{i,j}-X_{i,j+1}$. P is a projection operator to ensure $R_{i,j}^{2}+S_{i,j}^{2}\leq 1$, $\left|R_{i,n}\right|\leq 1$, and $\left|S_{m,j}\right|\leq 1$.

**Algorithm 2:** TV minimization.
**Input:** λ, Y, Vis, $\left(U^{1},V^{1}\right)=\left(R^{0},S^{0}\right)=\left(0,0\right)$, $t_{1}=1$, B=Y−Vis**Output:** 
X
1:**for**k=1**to***Maxiteration***do**;2:   $\left(R^{k},S^{k}\right)=P\left[\left(U^{k},V^{k}\right)+\frac{1}{8\lambda }L^{T}\left(B-\lambda L\left(U^{k},V^{k}\right)\right)\right]$;3:   $t_{k+1}=\left[1+\sqrt{1+4{\left(t_{k}\right)}^{2}}\right]/2$;4:   $\left(U^{k+1},V^{k+1}\right)=\left(R^{k},S^{k}\right)+\frac{t_{k}-1}{t_{k+1}}\left(R^{k}-R^{k-1},S^{k}-S^{k-1}\right)$;5:**end for**;6:$Z=B-\lambda L\left(R^{k},S^{k}\right)$;7:X=Z+Vis.


## 4. Experimental Results

In this section, we conduct experiments on publicly available datasets in comparison with several state-of-the-art methods to verify the effectiveness of our DRTV. We first introduce the testing data and compare methods and evaluation metrics. Then, we report the experimental results, followed by parameter analysis and computational cost comparison. All experiments are performed on a laptop with 1.9 GHz Intel Core i3 4030 CPU, 8 GB RAM, and Matlab code.

### 4.1. Dataset

The publicly available image dataset *TNO Human Factors*, which contains multispectral night images of different military scenarios, is used for performance evaluation. We select 20 infrared/visible images with different types of scenes for testing: *airplane_in_trees*, *helicopter*, *nato_camp_sequence*, *Farm*, *house_with_3_men*, *Kaptein_01*, *Kaptein_1123*, *Marne_02*, *Marne_03*, *Marne_11*, *Movie_01*, *Duine_7408*, *heather*, *house*, *Kaptein_1654*, *man_in_doorway*, *Movie_12*, *Movie_18*, *Movie_24*, and *Reek*.

Notably, visible and infrared images in the dataset are of the same resolution. As in real-world applications, the infrared images often present considerably low resolution. Here, we downsample the infrared images in our testing data for the experimental conditions to approximate the real-world situation. Therefore, all infrared source images present a low resolution (e.g., ratio *c* described in Section 2 is fixed to 2). We do not need image registration [37,38,39,40,41,42] before fusion because the source image pairs are all aligned.

We compare our DRTV with six state-of-the-art fusion methods, including pyramid transform-based methods such as Laplacian pyramid (LP) [43] and ratio of low-pass pyramid (RP) [44], multi-scale transform-based method such as HMSD [26]), edge-preserving decomposition-based method such as cross-bilateral filter (CBF) [45], subspace-based method such as directional discrete cosine transform and principal component analysis (DDCTPCA) [46], and total variation-based method such as gradient transfer fusion (GTF) [10]. Parameters in these methods are set by default as suggested in the original papers.

### 4.2. Evaluation Metrics

In numerous cases, the visual differences between fused results are insufficiently significant; therefore, fusion methods should be evaluated comprehensively from different perspectives. At present, evaluations are mainly divided into two directions: subjective evaluation and objective evaluation.

Subjective evaluation relies on human eyes and concentrates on the perception of details, contrast, sharpness, and target highlighting. While the quality of image is also affected by other factors, our eyes are insensitive to such factors. Numerous types of objective evaluation, which are mainly based on information theory, structural similarity, image gradients, and statistics, are available. Objective evaluation is consistent with human visual perception system and subject to experimental conditions.

The first two metrics, i.e., standard deviation (SD) [47] and entropy (EN) [48], are only dependent on the fused image. The three remaining metrics, i.e. mutual information (MI) [49], structural similarity index measure (SSIM) [50] and Peak signal-to-noise ratio (PSNR) [51], are determined by the fused and source images. In this paper, if the resolution of the fused image X is higher than that of the source image A (either the infrared image IR or the visible image Vis), then we must downsample the fused image first and then calculate the metrics.

(1) SD
  SD is a statistical concept reflecting distribution and contrast. SD is mathematically defined as follows:
  $$SD=\sqrt{\frac{1}{MN}\sum_{i=1}^{M}\sum_{j=1}^{N}{\left(X\left(i,j\right)-\mu \right)}^{2}},$$
  where μ is the mean value of X. Areas with high contrast are more likely to attract attention. Therefore, a large SD means that the fused image is improved.
(2) EN
  The EN of the fused image can measure the amount of information contained in it and is mathematically defined as:
  $$EN=-\sum_{l=0}^{L-1}p_{l}\log_{2}p_{l},$$
  where *L* is the number of gray levels and $p_{l}$ is the normalized histogram of the corresponding gray level. When the EN is large, additional information is contained in the fused image, and the performance of the fusion method is improved. However, EN is conventionally used as an auxiliary metric because it may be influenced by noise.
(3) MI
  MI is used to measure the amount of information transmitted from the source image to the fused image. MI is defined as follows:
  $$MI={MI}_{\mathrm{IR},X}+{MI}_{\mathrm{Vis},X},$$
  where ${MI}_{\mathrm{IR},X}$ and ${MI}_{\mathrm{Vis},X}$ respectively represent the amount of information transmitted from the infrared image and the visible image to the fused image. MI between two random variables can be calculated by the Kullback–Leibler metric:
  $${MI}_{A,X}=\sum_{a,x}p_{A,X}\left(a,x\right)\log_{2}\frac{p_{A,X}\left(a,x\right)}{p_{A}\left(a\right)p_{X}\left(x\right)},$$
  where $p_{A}\left(a\right)$ and $p_{X}\left(x\right)$ respectively represent the edge histograms of A and X. $p_{A,X}\left(a,x\right)$ is the joint histogram of A and X. A large MI metric means that considerable information is transferred from source images to the fused image, thereby indicating good fusion performance.
(4) SSIM
  To model image loss and distribution, SSIM was proposed by Wang et al. [52] as a universal image quality index based on structural similarity. The formula of the index is as follows:
  $${SSIM}_{A,X}=\sum_{a,x}b(a,x)\cdot c(a,x)\cdot s(a,x).$$
  In the formula above, to measure the structural similarity between source image A and fused image X, ${SSIM}_{A,X}$ is calculated and it mainly consists of three components: similarities of light, contrast and structure information, denoted as b(a,x), c(a,x) and s(a,x), respectively. The image patches of source image A and fused image X in a sliding window are respectively denoted by *a* and *x*. The specific definitions of the three components are as follows:
  $$b\left(a,x\right)=\frac{2\mu_{a}\mu_{x}+C_{1}}{\mu_{a}^{2}+\mu_{x}^{2}+C_{1}},$$
  $$c\left(a,x\right)=\frac{2\sigma_{a}\sigma_{x}+C_{2}}{\sigma_{a}^{2}+\sigma_{x}^{2}+C_{2}},$$
  $$s\left(a,x\right)=\frac{\sigma_{ax}+C_{3}}{\sigma_{a}\sigma_{x}+C_{3}},$$
  where $\mu_{a}$ and $\mu_{x}$ indicate the mean values of image patches *a* and *x*, respectively, reflecting brightness information; $\sigma_{a}$ and $\sigma_{x}$ represent SD, reflecting contrast information; $\sigma_{ax}$ denotes the covariance of source and fused images, reflecting the similarity of structure information; $C_{1}$, $C_{2}$, and $C_{3}$ are positive parameters used to avoid unstable phenomenon. Specifically, when $C_{1}=C_{2}=C_{3}=0$, the index is equivalent to the universal image quality index [53]. Thus, for all source and fused images, the index measure SSIM is defined as follows:
  $$SSIM=\omega_{Vis}{SSIM}_{\mathrm{Vis},X}+\omega_{IR}SSIM{,}_{\mathrm{IR},X},$$
  where ${SSIM}_{\mathrm{Vis},X}$ and ${SSIM}_{\mathrm{IR},X}$ indicate structural similarities between visible/infrared and fused images, respectively.
(5) PSNR
  PSNR is used to reflect the distortion by the fusion operation and is defined by the ratio of peak value power and noise power in the generated fused image:
  $$PSNR=10\log_{10}\frac{r^{2}}{MSE},$$
  where *r* denotes the peak value of the fused image and MSE denotes the the mean square error between the fused image and source images. The larger the PSNR, the less distortion the fusion method produces.

### 4.3. Results and Analysis

To maintain a considerable amount of information in source images, we select the strategy of upsampling infrared images rather than downsampling visible images for the comparison methods before fusion, that is, e.g., the procedure (c) in Figure 1. Our DRTV involves only one parameter λ, which we set to 50 as its default value.

**Figure 2:**
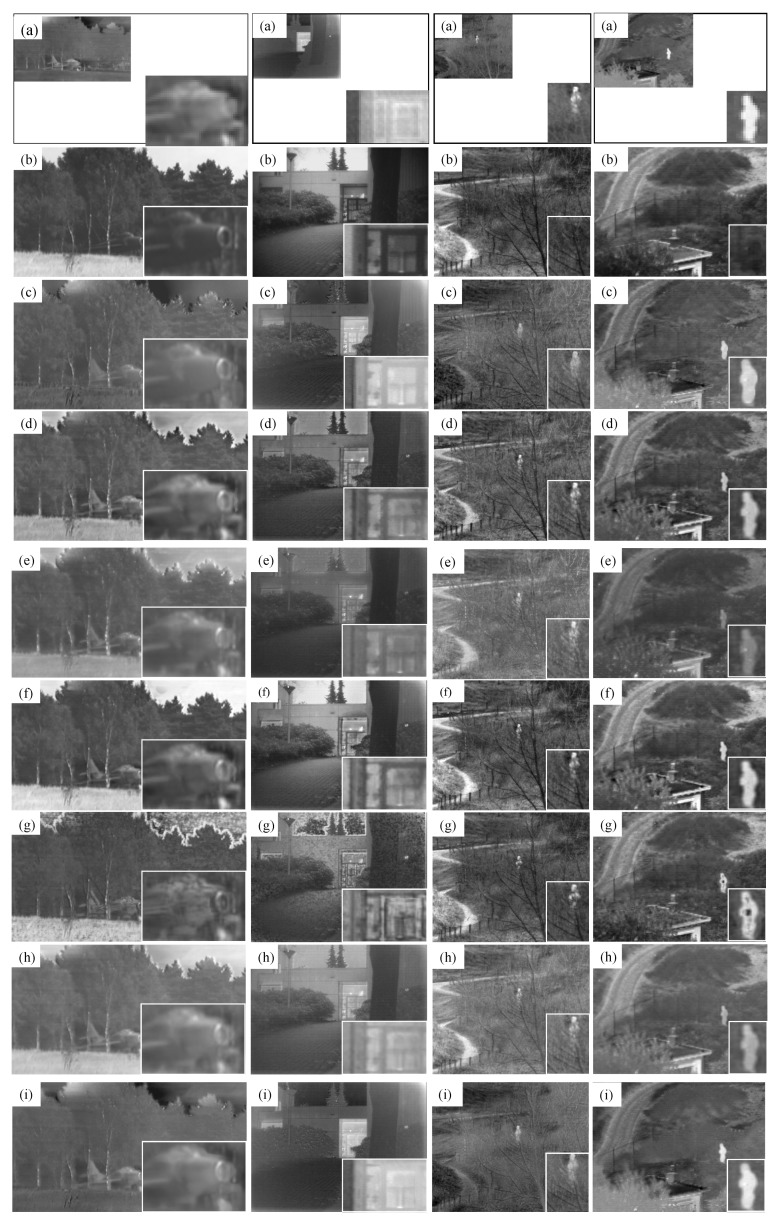
Fusion results of different fusion methods on four image pairs. The four groups of results from left to right: *airplane_in_trees*, *Kaptein_01*, *nato_camp_sequence* and *sandpath*, where the corresponding subfigures are: (**a**) infrared image; (**b**) visible image; (**c**) our DRTV; (**d**) LP [43]; (**e**) RP [44]; (**f**) HMSD [26]; (**g**) CBF [45]; (**h**) DDCTPCA [46]; (**i**) GTF [10].

We first conducted several qualitative comparisons of different methods on four typical image pairs, including *airplane_in_trees*, *Kaptein_01*, *nato_camp_sequence*, and *sandpath*, as shown in Figure 2. Compared with other six fusion methods, DRTV considerably improves the resolution of the fused images and does not suffer from strong noise caused by upsampling infrared images before fusion. Although the resolution of infrared images is only half of that of visible images, DRTV allows preservation of additional detailed texture information, which benefits from the downsampling operator in $\varepsilon_{1}\left(X\right)$ and the ${∥\cdot ∥}_{TV_{I}}$ norm of gradient differences in $\varepsilon_{2}\left(X\right)$, in visible images. Furthermore, the fused results generated by our DRTV are similar to high-resolution infrared images with detailed scene representations, which are beneficial for detecting and recognizing potential targets.

**Figure 3:**
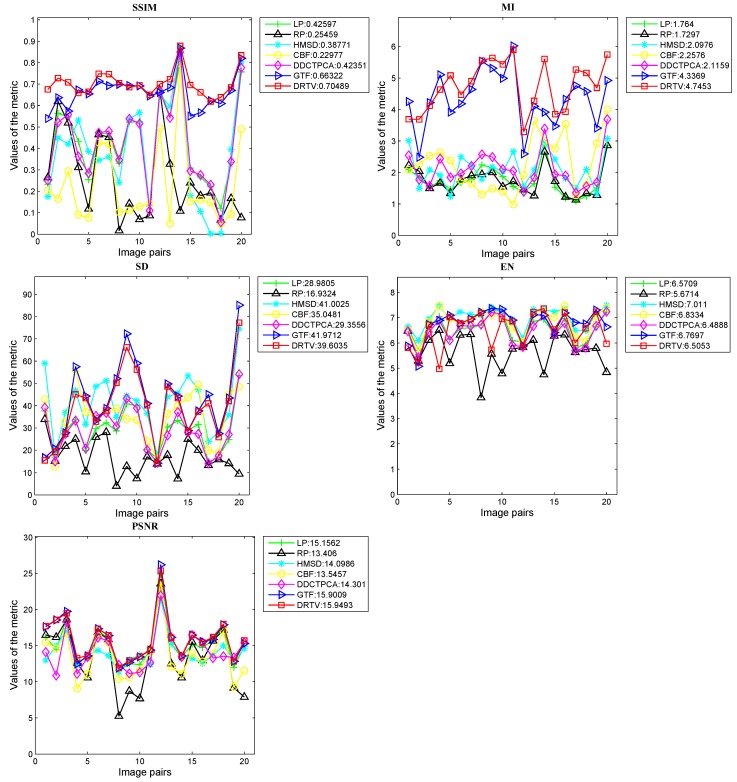
Quantitative comparisons of six fusion methods on twenty image pairs.

Next, we provide quantitative comparisons on the total dataset with the following five metrics described in the last section: SSIM, MI, SD, EN and PSNR. The results are shown in Figure 3. For the metrics SSIM, MI and PSNR, our DRTV can generate the largest average values and exhibits the best performance on 17, 15 and 9 image pairs, respectively. For the metric SD, DRTV can produce comparable results. Meanwhile, our method for the metric EN does not achieve the best results, which can be explained as follows. Entropy is defined as a measure of uncertainty. When the probability of each gray value is evenly distributed, the uncertainty and the entropy are both large. For our DRTV, we constrain the fused image and the infrared image to present similar intensity distribution to facilitate discrimination of the thermal target by human eyes. Therefore, our fusion result maintained the intensity distribution of the original infrared image, where the gray value is unevenly distributed as that in the visible image, leading to a small EN value.

### 4.4. Parameter Analysis

**Figure 4:**
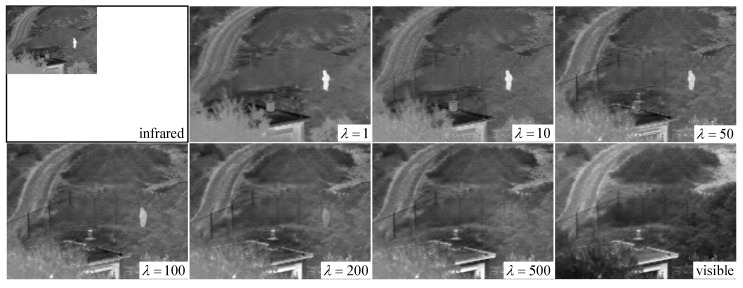
Fusion results of our DRTV on *nato_camp_sequence* when parameter λ increases.

**Figure 5:**
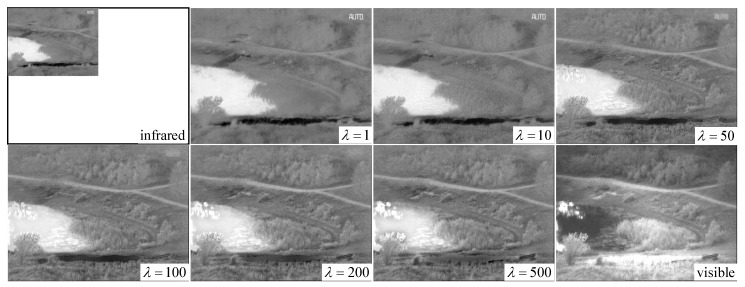
Fusion results of our DRTV on *lake* when parameter λ increases.

Only one parameter is presented in our proposed method, i.e., the regularized parameter λ, which controls the trade-off between $\varepsilon_{1}\left(X\right)$ and $\varepsilon_{2}\left(X\right)$. When λ is small, the fused image preserves additional thermal radiation information in the infrared image, allowing the visual effect to be highly similar to the infrared image. Conversely, when λ is sufficiently large, the texture information in the visible image remains in the fused image to a large extent; hence, the fused image is similar to the visible image. When λ→+∞, the fused image is the original visible image. To analyze the influence of parameter λ on fusion performance in detail, we test different values of λ on two image pairs, including *nato_camp_sequence* and *lake*. We gradually increase the value of λ and observe the changes of the fused images. Figure 4 and Figure 5 show the changes of the fused images of the two image pairs when λ is fixed to 1, 10, 50, 100, 200, and 500.

As λ changes from 1 to 500, the fence and hill around the house in Figure 4 gradually appear. In addition, the outline of the chimney in the bottom left corner becomes gradually clear. For the results in Figure 5, the most evident change is that the texture of vegetation on the river is clearly delineated, and the chair is gradually separated from the road. Thus, we can conclude that texture features in the fused image gradually become clear as the value of λ increases.

By contrast, we can observe that, in Figure 5, when λ is smaller than 200, the pedestrian in *nato_camp_sequence* is legible, and the gray distribution of the house in the bottom left corner is similar to that in the infrared image. Meanwhile, when λ is set as 500, the pedestrian’s thermal radiation information is completely lost in the fused image and the house is similar to that in the visible image. In Figure 5, the word “AUTO” in the upper right corner gradually disappears in the fused image as λ changes from 1 to 500. Therefore, we conclude that, as λ increases, thermal radiation information becomes gradually lost in the fused image. This finding is also the flexibility and advantage offered by our method. By adjusting the value of parameter λ, users can conveniently control the similarity of the fused image to the infrared image or the visible image.

### 4.5. Computational Cost Comparison

**Figure 6:**
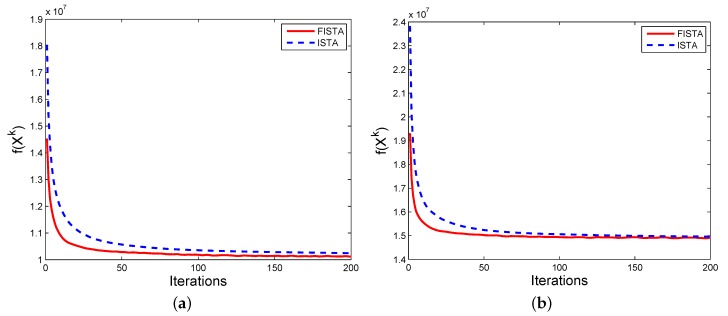
Convergence rate comparison between FISTA framework and previous variational model. ((**a**) result on *airplane_in_trees*; (**b**) result on *Kaptein_01*).

Given that the FISTA framework is introduced in our model to accelerate the convergence rate, to evaluate it, we compare the model solved by FISTA framework and that solved by the previous variational model, ISTA. The convergence rate is observed through the value of $f\left(X^{k}\right)$. The objective function $f\left(X\right)$ is denoted by $\varepsilon \left(X\right)$ in Equation (8) and $X^{k}$ denotes the fused image obtained by the *k*-th iteration. Figure 6 demonstrates the convergence rate comparison corresponding to two image pairs in Figure 2, e.g., *airplane* and *Kaptein_01*, as an example. Inheriting the advantage of the FISTA framework, our method typically converges only after 20 to 50 iterations while the method based on ISTA often converges in 70 to 90 iterations. Additional iterations will increase the computational cost without substantial performance gain. To stop the iteration automatically by the value of $f\left(X^{k}\right)$, we apply the residual ratio of $f\left(X^{k}\right)$ to stop the iteration process. The residual ratio (RR) is defined as:

(14) $$RR=\frac{\left|f\left(X^{k}\right)-f\left(X^{k-1}\right)\right|}{f\left(X^{k-1}\right)}.$$

During the iteration process, the iteration is stopped when the value of RR is less than a predefined threshold and in our method, and the threshold is set as 0.3% empirically.

**Figure 7:**
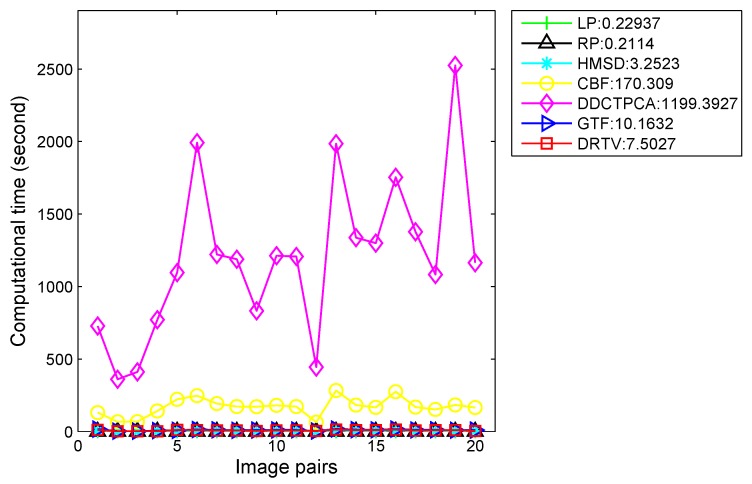
Runtime comparison of different methods on the whole test dataset.

To evaluate the efficiency of our proposed method, we compare our method with six other methods in terms of runtime. The computational costs of seven methods on 20 image pairs are shown in Figure 7, and the average runtime of seven algorithms are reported in the legend. In DDCTPCA, the source images are divided into many non-overlapping square blocks and there are eight directional modes to be performed on each block. All the coefficients in eight modes are used for fusion. Thus, the process of obtaining and fusing the coefficients for all the blocks takes up most of the runtime. Therefore, the runtime has a lot to do with the number of blocks, i.e., the size of source images. This is the reason why the runtime of DDCTPCA is oscillating, while, in our method, we formulate the problem as a convex optimization problem characterized by total variation model and sizes of source images have less effects on computational cost. This finding demonstrates that our method can achieve comparable efficiency.

## 5. Conclusions and Future Work

In this paper, we proposed a method for fusing infrared and visible images of different resolutions called DRTV. The fusion process is similar to conducting infrared image super-resolution, which simultaneously integrates the texture detail information in the visible image. The quantitative comparisons on several metrics with six state-of-the-art fusion methods demonstrate that our method can not only keep more detailed information in the source images, but also considerably reduce noise in the results caused by upsampling infrared images before fusion which typically occurs in existing methods. The fused image can also retain the thermal radiation information to a large extent, thereby benefiting fusion-based target detection and recognition systems.

In our objective function, we have used the first-order TV to preserve the texture detail information in the visible image. The first-order TV performs well in preserving edges of object in the piecewise constant image compared with other total variational models. However, it will produce staircase effects. For the purpose of eliminating the staircase effect in the first-order TV model, high-order models have been proposed by modifying the TV regularizer in the first order variational model to other regularizers and have shown promising performance, e.g., the TGV regularizer in [54], the bounded Hessian regularizer together with an edge diffusivity function in [55], Laplacian regularizer and regularizers combining the aforementioned regularizers with the TV regularizer [56]. Thus, it deserves attention to modify our model with high-order variational models to remove staircase effects in the follow-up work.
